# The General Meetings of the British Medical Association

**Published:** 1899-10-28

**Authors:** 


### THE GENERAL MEETINGS OF THE BRITISH MEDICAL ASSOCIATION.

To those members of the British Medical Association who felt themselves called upon to waste their time and spend their energies in passing resolutions down at Portsmouth, it must be rather disappointing to find from the report of the proceedings of the Council of the Association what has been the fate of their pronouncements. In the majority of cases when these resolutions came up for consideration by the Council it was resolved that they "lie on the table." And there they lie; hibernating on the office table in the Strand until perchance the warmth of some future general meeting shall enable them to flutter for a few hours once again.

The resolution on the Midwives' Bill, the censure on Sir William Priestley, the condemnation of the teaching of midwifery and cognate subjects to other than recognised medical students, the resolution on registration of qualified practitioners in India, the censure upon the President of the Council and upon the Council itself for burking the discussion upon the expenditure on the journal, the attack on their editor, and the resolution in regard to medical defence and the maintenance of the honour of the profession, all these matters, which had been discussed with so much energy, and on which such very definite resolutions had been passed by the Association in general meeting assembled, were quietly shelved by the Council by the simple method of resolving that they "lie on the table."

Not that we think the Council is wrong. The waste-paper basket would probably have been even more appropriate a resting place. But is it not time that an end should be put to this ridiculous annual performance? The British Medical Association gives itself out to be the greatest medical society in the world, and perhaps it is. Its annual meetings certainly pose as being something far more important than mere gatherings of excursionists, and that they are accepted at their own valuation is shown by the splendid hospitality with which they are everywhere received. From all parts of the earth men gather together, and, using the machinery provided by the laws of the Association, pass ponderous resolutions on current events. The world is imagined to tremble at this organised voice of a united profession. The world perhaps, but not the Council; for the Council, knowing how these theatrical thunderings are made, is not so easily startled from its serenity.

It seems to us that the first duty of the Association is either to put a stop to the farcical proceedings at these general meetings, or to render them so representative that their resolves shall of necessity lead to action. The present position is derogatory to the dignity of the profession.

